# Spontaneous colloidal metal network formation driven by molten salt electrolysis

**DOI:** 10.1038/s41598-018-31521-3

**Published:** 2018-08-30

**Authors:** Shungo Natsui, Takuya Sudo, Takumi Kaneko, Kazui Tonya, Daiki Nakajima, Tatsuya Kikuchi, Ryosuke O. Suzuki

**Affiliations:** Division of Materials Science and Engineering, Faculty of Engineering, Hokkaido University Kita 13 Nishi 8, Kita-ku, Sapporo, 060-8628 Japan

## Abstract

The molten salt-based direct reduction process for reactive solid metal outperforms traditional pyrometallurgical methods in energy efficiency. However, the simplity and rapidity of this process require a deeper understanding of the interfacial morphology in the vicinity of liquid metal deposited at the cathode. For the first time, here we report the time change of electrode surface on the sub-millisecond/micrometre scale in molten LiCl-CaCl_2_ at 823 K. When the potential was applied, liquid Li-Ca alloy droplets grew on the electrode, and the black colloidal metal moved on the electrode surface to form a network structure. The unit cell size of the network and the number density of droplets were found to depend on the applied potential. These results will provide important information about the microscale mixing action near the electrode, and accelerate the development of metallothermic reduction of oxides.

## Introduction

Direct electrochemical reduction of solid oxides (XO_x_, X = Ti, Zr, Hf, V, Nb, Ta, U, and other rare dispersed metals) in molten chloride (e.g. CaCl_2_, LiCl, KCl, NaCl, and their mixtures) is a simple and straightforward electrolytic metallurgical method, which outperforms traditional pyrometallurgical methods such as carbothermic and metallothermic reductions in terms of energy efficiency. Among the present systems of titanium metal production^1^, the direct electrochemical decomposition of TiO_2_ in molten CaCl_2_ or LiCl has received special attention because of its simplity and low energy cost. (For comparison, in the prevailing Kroll process, TiO_2_ must be converted to TiCl_4_ by Cl_2_ gas beforehand. Then, liquid Mg is used as the reductant, and MgCl_2_ as a by-product is circulated by electrolysis to give liquid Mg and gaseous Cl_2_.) One successful example is the widely known “FFC Cambridge process”, in which the oxide anion from the solid TiO_2_ pellet placed at the cathode transfers to the anode in the salt bath^2–5^. Because the Ti-O binary system contains many lower oxides than TiO_2_, oxygen in a higher oxide is removed to form a lower oxide TiO_y_(y < 2) upon receiving electrical charge from the cathode. For higher productivity, another promising method (“OS process”) has been proposed that has better utilisation of the oxide anion transfer in CaCl_2_, because as much as 20 mol% CaO can dissolve in molten CaCl_2_ at 1173 K^6–12^. The electrochemically deposited liquid Ca at the cathode also dissolves in the CaCl_2_ melt (the solubility of Ca is reported as 2–4 mol% at 1123 to 1198 K)^13,14^; and the dissolved Ca works effectively to reduce the oxide powder, even if the powder particles are electrical insulators or have no direct electrical contact with the cathode. Similarly, LiCl and its binary chloride systems can dissolve oxygen anion at lower temperatures^15–17^. Moreover, the LiCl-CaCl_2_ eutectic melt operates at a low temperature compared with simple salts (sometimes KCl is added for further lower the temperature), so it is attracting attention as a molten salt with high energy efficiency and high reducing properties^18–21^.

In the mechanism of the OS process, metallothermic reduction by the dissolved Ca in the vicinity of the cathode is essential, where the oxide is placed close to the cathode. In the case of metallothermic reduction of solid oxide XO_x_(s) using electrodeposited liquid metal (Me = Ca, Li, or their alloys), the morphology of Me near the cathode is crucial when the oxide is reduced in the following mechanism^8,9,12,22^:

1 $${\rm{MeO}}\to {{\rm{Me}}}^{2+}+{{\rm{O}}}^{2-}$$

2 $${{\rm{Me}}}^{2+}+2{e}^{-}\to \mathrm{Me}\,({\rm{vicinity}}\,{\rm{of}}\,{\rm{the}}\,{\rm{cathode}})$$

3 $${{\rm{XO}}}_{{\rm{x}}}({\rm{s}})+{\rm{Me}}\to {{\rm{XO}}}_{{\rm{x}}-1}\,({\rm{s}})+{\rm{MeO}}$$

where the liquid Me works effectively to reduce XO_x_, even though the latter does not have any direct electrical contact with the cathode. Therefore, the reduction efficiency in this method is thought to be greatly affected by the morphology of the interface between the electrolytically deposited Me and molten salt. It is widely known that a “metal fog” (colloidal metal) could be formed around the deposited Me without mechanical stirring, if a suitable emulsifier is present^23–27^. Electrochemically deposited colloidal Me in molten salt has been considered to be particles formed by Me_x_ molecular clusters^28–30^. Despite its importance, however, there is only limited knowledge about the behaviour of colloidal Me due to the difficulty of *in-situ* observation^31–37^. Recently, black filmlike colloidal Me was observed on an electrodeposited thin Li metal in molten LiCl-KCl^38^; however, the detailed mechanism of colloidal Me flow has not been clarified. Although colloidal Me could be generated at the liquid Me-molten salt interface, its morphology on the electrochemically precipitated cathode surface (which is the root cause of such phenomenon) at the sub-millisecond/micrometre scale remains poorly understood for high-temperature molten salts.

A clear understanding of the behaviour of colloidal Me is necessary for control and optimisation of the convection field near the electrode. Besides the FFC and OS processes, such knowledge can be applied immediately to the current molten salt electrolysis process, and brings about large energy saving from the viewpoint of thermal efficiency in the material industry. Meanwhile, high-speed microscopy at the resolution of low-magnification scanning electron microscopes has been made possible by recent complementary metal oxide semiconductor-based image sensors, which have been improved by progresses in digital optical technology. In this study, we investigated the dispersion characteristics of colloidal Me in molten LiCl-CaCl_2_ by examining images of the electrode surface obtained with high-speed digital microscopy synchronised to the electrochemical measurement.

## Results

**Figure 1:**
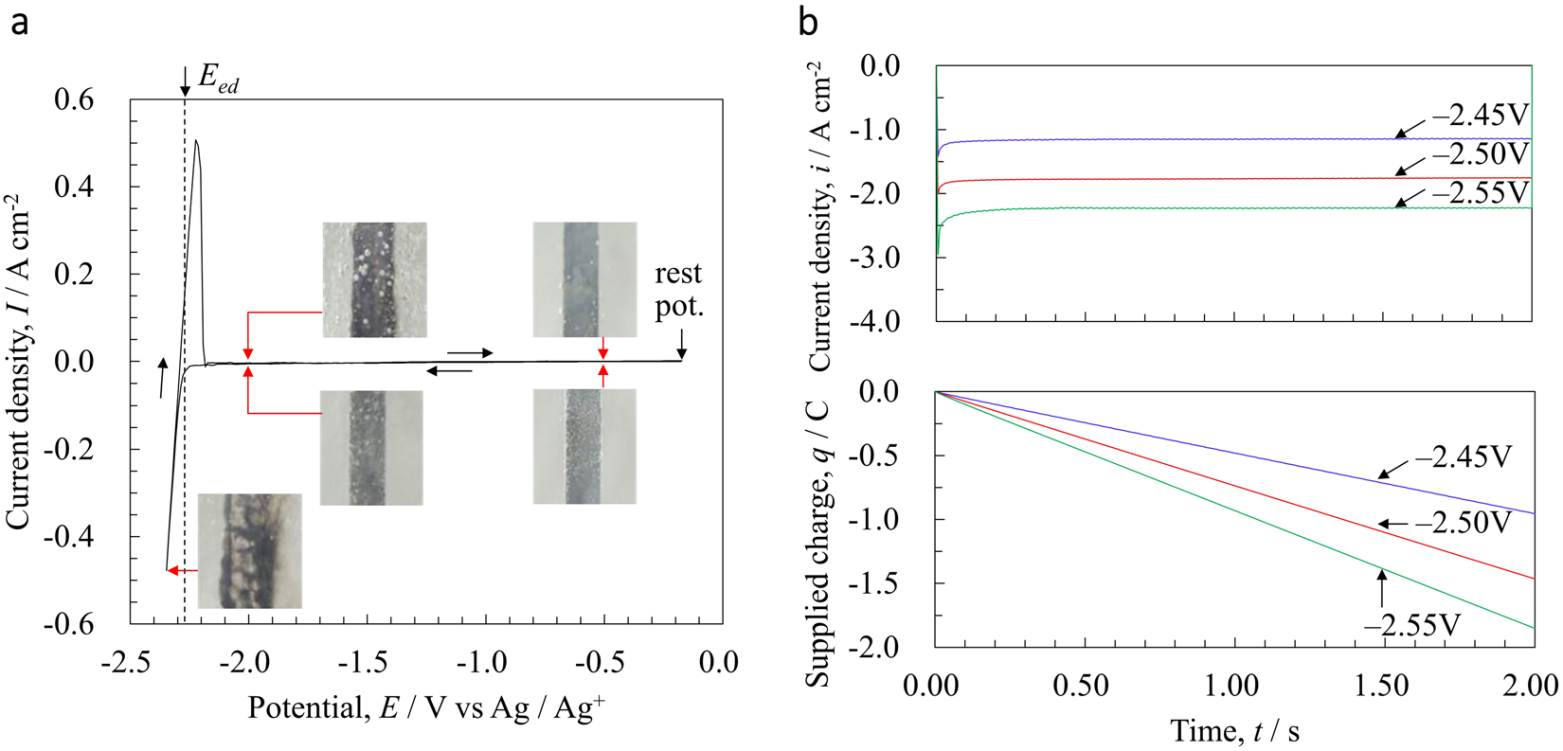
Electrochemical characteristics of the Mo electrode in LiCl-CaCl_2_ eutectic melt at 823 K (**a**) Cyclic voltammogram at the scan rate of 10 mV/s, together with representative photographed working electrode(WE) (*ϕ*1.5 mm) images at different potentials captured using a single-lens reflex digital camera (D810, Nikon Co.) (**b**) Time change of current density and supplied charge in each potential condition derived by chronoamperometry.

**Figure 2:**
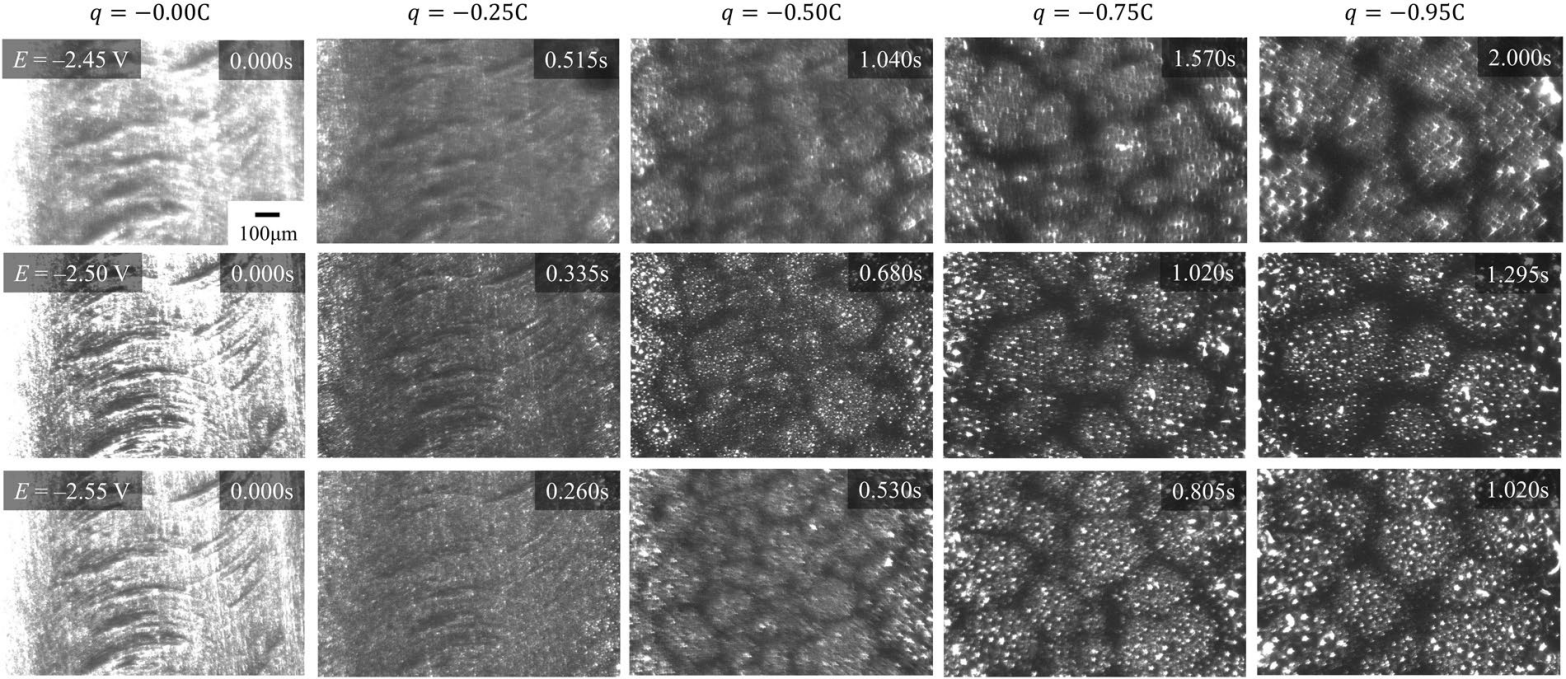
Photographs of the electrodeposited melt and black colloidal metal formed on the flat Mo electrode at 823 K. The snapshots correspond to almost the same region. The corresponding current-time curves are represented in Fig. 1b. (In Supplementary Video, we reported the temporal change of the electrode surface at *E* = −2.55 V.)

Figure 1 shows the measured electrochemical data for the Mo electrode in LiCl-CaCl_2_ eutectic melt at 823 K. The cyclic voltammograms are shown in Fig. 1a, and a sharp increase in the cathodic current was observed at about ${E}_{ed}=-\,2.285\,{\rm{V}}$ versus Ag^+^/Ag, as well as in the corresponding anodic current. These currents are thought to be due to the deposition and dissolution of Li-Ca alloy, respectively^20,21^. Using thermodynamic analysis of the free energy change and quantitative analysis of the electrodeposited samples, we determined the approximate composition of the electrodeposited alloy at this peak to be Li: Ca = 71: 29 (Appendix 1^39,40^ and Appendix 2^41–44^). The ratio between the electric charges passing through the anode and the cathode in the voltammograms (${q}_{a}/{q}_{c}$) gives a momentary current efficiency of ${q}_{a}/{q}_{c}=0.904$. In the snapshots taken at different potentials, black colloidal Me was observed with the electrodeposited Li-Ca alloy. The reason for ${q}_{a}/{q}_{c} < 1$ must be due to the diffusion of colloidal Me, especially by chemical dissolution. At $E < {E}_{ed}$, the surface of the electrode was shiny metallic, while colloidal Me exists in the vicinity of interface between salt and bulk liquid Li-Ca alloy. The time changes in current density and supplied charges at several electrochemical potentials are shown in Fig. 1b. These C-T curves represent the chronoamperogram conducted at −2.45, −2.50, or −2.55 V vs. Ag^+^/Ag for 2.0 s. Non-faradaic current corresponding to electric double layer formation was found in the early stage of electrolysis. When *t* > 0.02 s, the current density was nearly constant, which is consistent with the previous study^21^. Namely, the electrolytic deposition reaction proceeded at a constant rate. Photographs of the electrode surface along the current-time curves are shown in Fig. 2. In contrast to the C-T curves, the electrode interfaces showed rather complicated morphology at this scale. At any potential, the liquid-phase precipitate grew heterogeneously on the flat Mo surface, eventually generating a huge number of metal droplets. In contrast, colloidal Me was generated around the electrode. We discovered that colloidal Me moves near the electrode surface to form a “network”. This network can be considered to be 2-dimensional with “unit cells” whose diameter tends to increase with time. After prolonged electrolysis, the cell structure collapsed due to excessive precipitation of colloidal Me and aggregation of the network structure (Appendix 3). When the potential is more negative, the cells are larger for the same supplied charge, possibly because the convection field is generated more rapidly. Further, a more negative potential promotes the aggregation of colloidal Me, and droplets can be clearly seen at the central portion of the cells.

## Discussion

**Figure 3:**
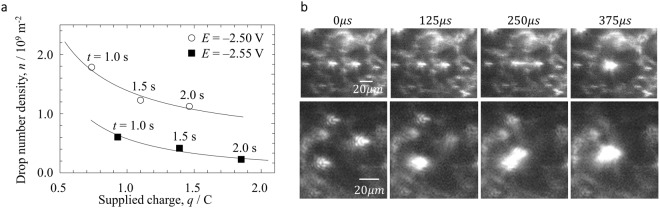
Morphology of metal droplets electrolytically deposited on flat Mo surface (**a**) Potential-dependent temporal changes of the droplet number density. The number of droplets in areas of 1.28 × 10^−7^ m^2^ was counted using ImageJ, and the average value thereof was calculated (**b**) Representative coalescence behaviour between electrodeposited droplets (*t* > 1.0 s, *E* = −2.55 V).

We investigated the correlation between the morphology of Ca-Li alloy droplets electrodeposited on the Mo electrode surface and the condensation behaviour of colloidal Me in the vicinity. The relationship between the supplied charge and drop number density (at least for the obvious droplets at *t* > 1.0 s) is shown as Fig. 3a. As the amount of supplied charge increased, the droplets coalesced repeatedly, thus their number density decreased. A lower droplet number density was found at *E* = −2.55 V than at −2.50 V, meaning that larger droplets were formed at the more negative potential. Supplying the same electric charge (*q* = −0.95 C) took less time at *E* = −2.55 V than at −2.50 V. As shown in Fig. 3b, the coalescence process involving 2 or 3 droplets was completed on a time scale of *t* < 500 μs, so we can exclude the coalescence speed as a factor. Therefore, it is natural to consider that the static droplet diameter changes due to the effect of the potential on the interfacial tension balance (electric capillary phenomenon)^45^.

The electrodeposited colloidal Me should form a “diffuse electric double layer” due to the surface charge of the colloid. Then, van der Waals and electrostatic interactions should occur spontaneously to maintain electrical neutrality based on the well-known Derjaguin-Landau-Verwey-Overbeek (DLVO) theory^46^. Moreover, interactions other than such potential forces should also be considered for the formation of a stable network structure, because it was pointed out that hydrodynamic interaction affects the structure of colloidal networks^47,48^. After the network structure is formed, it has to be partially distorted for the coarsening of the cohesive structure. Although colloidal particles generally aggregate to minimize the local cohesive energy, this distortion process must also increase the local cohesive energy. Since the network aggregation structure has a tendency to shrink as a whole system, the spontaneously generated stress is stored instead. Thus, given the energy conservation, the hydrodynamic driving force must create distortion in the colloidal metal network. In the present system, this can be driven by a sharp gradient in temperature or concentration due to the electrochemical reaction (Appendix 4)^49–52^.

**Figure 4:**
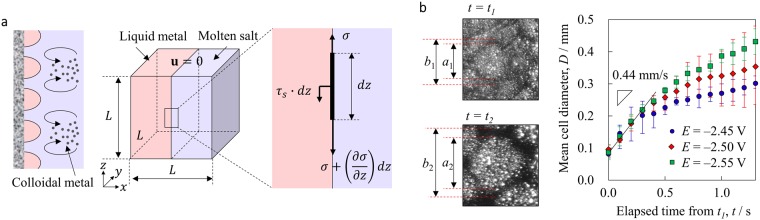
Cell generation mechanism by interfacial tension gradient-induced convection. (**a**) Schematic diagram of Marangoni convection near electrodes. (**b**) Length and grow rate of cells in the colloidal network obtained from image analysis. The minimum and maximum diameters of a given cell are denoted respectively as *a* and *b* in consideration of the thickness of the network, and the mean cell diameter is given based on multiple cells. *t*_1_ is the reference time when the cell was formed.

The famous cellular convection structure called “Bénard cells” is formed in the vertical direction driven by buoyancy. Block confirmed that a polygonal cell convection pattern can be generated in the liquid phase with thickness ≤1 mm in any direction (not just vertically)^53^. This flow mechanism of two liquid phases is known as “Marangoni-Bénard convection”. Generally, this flow is generated by the attractive force from the low interfacial tension region to the high interfacial tension region as the driving force (the Marangoni effect). In this case, it is known that the liquid level at the centre of the cell is lower than around the cell^54^. Although high-temperature melts generally have high interfacial tension, it was estimated that the interfacial tension between liquid Me and molten salt during electrolysis is lower than many room-temperature systems, e.g. water-oil. Also, because it is a very unstable interface, interfacial tension gradient can easily occur there (Appendix 2)^55,56^. For the flow generated at the liquid Me-molten salt interface, the mechanism discussed above is schematically shown in Fig. 4a. Let us assume a constant interfacial tension gradient and two liquid phases (electrodeposited liquid metal and molten salt) on the Mo electrode (solid phase). A flow should occur when the liquid phases with different interfacial tensions are brought into contact with each other on the solid phase. Non-slip boundary condition is set on both end faces of the *x*-*z* plane, and velocity in the *x* direction for the upper face of the *x*-*y* plane is set as ${\bf{u}}=0$. When $L$ is the length of the representative cube around the solid-liquid-liquid interface, the Marangoni force can be simply estimated as ${{\bf{F}}}_{m}={\rm{\Delta }}\sigma L$. Here, ${\rm{\Delta }}\sigma$ is the interfacial tension gradient due to temperature or concentration gradient or both, as mentioned above. When ${{\bf{F}}}_{m}$ and the shear forces on the *x*-*y* and *x*-*z* planes (the surface area should be ${L}^{2}$ in each) are balanced, the flow will reach steady state. The shear force can be estimated from Newton’s law of viscosity. When a maximum velocity ${{\bf{u}}}_{m}$ is reached at the bottom centre of the cube, the shear stress on the *x*-*y* plane (${\rm{\Delta }}\sigma L-\mu \frac{{{\bf{u}}}_{m}}{L}{L}^{2}$) and that on the *x*-*z* plane ($-\mu \frac{4{{\bf{u}}}_{m}}{L}{L}^{2}$) are equal, and we get ${{\bf{u}}}_{m}={\rm{\Delta }}\sigma /5\mu$. Here, the viscosity coefficient of the molten salt was estimated as $\mu =5\times {10}^{-3}\,\mathrm{Pa}\cdot {\rm{s}}$^44^. The typical growth rate of the cells was 0.44 mm/s based on the time change of the cell diameter obtained in the experiment, as shown in Fig. 4b. Our model estimates the interfacial tension gradient to be ${\rm{\Delta }}\sigma =0.1\,\mathrm{mN}/{\rm{m}}$ for the flow velocity ${{\bf{u}}}_{m}=0.4\,{\rm{mm}}/{\rm{s}}$. It is suggested that this slight interfacial tension difference causes the flow velocity observed in this system. Such a flow induced by the interfacial tension difference between electrodeposited Me and molten salts may promote the electrochemical reaction. This discovery provides a new perspective to improve the efficiency of the OS process, by adding substances that promote surface activity and searching for suitable electrolysis conditions.

## Conclusions

We visualised the convection field with a colloidal network generated at the Mo electrode interface during electrolysis of CaCl_2_-LiCl eutectic melt at 823 K. When a potential was applied, growth of liquid metal droplets on the electrode was observed, and a black colloidal metal moved on the electrode surface to form a network structure. The determined dependence of unit cell size of the network on the applied potential will make a significant contribution to high-temperature colloid chemistry in the future. Simultaneously, using a simplified hydrodynamic model, a relatively large flow velocity is expected to occur on this temporal and spatial scale even with a slight interfacial tension difference. This non-uniform interfacial flow caused by heat and mass transfer will make an effective contribution to the microscale mixing in the vicinity of the electrode.

## Method

To elucidate the interfacial morphology of the working electrode, a quartz glass vessel 100 mm in diameter and 250 mm in height (Kondo Science, Inc.) was employed, which has a barrel-vaulted shape with a flat surface for *in-situ* observations. An electric resistance furnace (SiC heater) was designed to control the inner vessel temperature with an accuracy of ±1 K for direct observation of processes within the vessel. A metal halide light (HVC-SL, maximum light flux: 12,500 lm, main spectral peak: 550 nm) was used as an auxiliary light source. Changes in the electrode interface were recorded at a rate of 8000 fps (125 μs intervals), and a resolution of 640 × 480 pixels was obtained using a high-speed digital camera (Ditect Co., Ltd., HAS-D71, monochrome, main response spectral range: 500–600 nm) and a long-distance zoom lens (VS Technology Co. Ltd., VSZ-10100, working distance: 95 mm, minimum field of view: 666 μm × 500 μm, and length per pixel: 1.04 μm). The location of the tip of the liquid metal-electrode interface was tracked in each captured image by using image processing software (Photron Co., Ltd., PFV Viewer and ImageJ). Reagent-grade LiCl (Wako Pure Chemical Co. Ltd., >99.0%) and CaCl_2_ (Wako, >95%) were used for the melt. The eutectic mixture of LiCl-CaCl_2_ (65:35 mol%, m.p. = 748 K, 720 g) in a borosilicate glass crucible with flat surface was dried under approximately 1 Pa at 573 K for more than 12 h. Then, it was heated to 823 K (the constant experimental temperature) and kept for 5 h to remove residual water. All the experiments were conducted in an Ar atmosphere (>99.9995%). The melt temperature was measured with a K-type thermocouple with a glass protection tube. After melting the mixed salt, the suspended electrodes were immersed in the melt while keeping the seal. The working electrode was a Mo rod (Nilaco Corp., ϕ 1.5 mm, 99.95%) which was previously trimmed flatly in half by end-milling on the observation side, and the rod surface was polished with emery paper. The immersion depth of the working electrode was fixed at 10 mm by using an insulated protective Al_2_O_3_ tube. The counter electrode was a graphite rod (Toyo Carbon Corp., ϕ 10 mm). An Ag^+^/Ag reference electrode was employed, which consisted of a silver wire (ϕ 1.0 mm, 99.99%, Nilaco) immersed in a LiCl-CaCl_2_ eutectic melt containing 0.5 mol% AgCl (Wako, 99.5%) and set in a borosilicate tube^20^. Electrochemical measurements were performed using an automatic polarisation system (Hokuto Denko Corp., HZ-5000). The inter-electrode voltage and microscope images were synchronised with an error of 4 μs by using an analogue signal synchronous system (Ditect Co., Ltd., DI-SYNC 29 N). A schematic diagram of the experimental apparatus is depicted in Appendix 5.

## Electronic supplementary material

Supplementary information

Temporal change of the electrode surface at E = −2.55 V. (playback speed: 800 fps)
