# Supplementary material for: Spontaneous colloidal metal network formation driven by molten salt electrolysis
Source: Sci Rep. 2018 Aug 30;8:13114. doi: 10.1038/s41598-018-31521-3 (PMC6117334; doi:10.1038/s41598-018-31521-3)
Supplement: Supplementary file 1 — Supplementary information [file 41598_2018_31521_MOESM1_ESM.pdf]

Supplementary Information

Spontaneous colloidal metal network formation driven by molten salt electrolysis

Shungo Natsui,* Takuya Sudo, Takumi Kaneko, Kazui Tonya,
Daiki Nakajima, Tatsuya Kikuchi, and Ryosuke O. Suzuki

Division of Materials Science and Engineering, Faculty of Engineering,
Hokkaido University
Kita 13 Nishi 8, Kita–ku, Sapporo, 060–8628 Japan.

*Corresponding author. Tel.: +81–11–706–6342
E–mail address: natsui@eng.hokudai.ac.jp

Appendix 1. Deposition potential of Li–Ca alloy

Consider the metal species Me and the electrolysis of its chloride $MeCl_2$. If two–electron reaction occurs with chlorine evolution at the anode, the equilibrium potential is given by the Nernst equation as follows.

$$\mathrm{Me}^{2+} + 2\mathrm{e}^{-} \rightarrow \mathrm{Me}\ (\text{at cathode}) \quad \text{(s1)}$$

$$2\mathrm{Cl}^{-} \rightarrow \mathrm{Cl}_2 + 2\mathrm{e}^{-}\ (\text{at anode}) \quad \text{(s2)}$$

$$E_1 = E^0_{\mathrm{Me}} + \frac{RT}{2F}\ln\frac{{a_{\mathrm{Me}^{+}}}^2}{{a_{\mathrm{Me}}}^2} \quad \text{(s3)}$$

$$E_2 = E^0_{\mathrm{Cl}} + \frac{RT}{2F}\ln\frac{{a_{\mathrm{Cl}^{-}}}^2}{P_{\mathrm{Cl}_2}} \quad \text{(s4)}$$

where E is the electrochemical potential, E^0 is the standard electrode potential, R is gas constant, T is temperature, F is Faraday constant, a is the activity, and P is the partial pressure. When the Ag/AgCl reference electrode is used for this electrochemical system, we also have

$$2\mathrm{Ag}^{+} + 2\mathrm{e}^{-} \rightarrow 2\mathrm{Ag}\ (\text{at RE}) \quad \text{(s5)}$$

$$2\mathrm{Cl}^{-} \rightarrow \mathrm{Cl}_2 + 2\mathrm{e}^{-}\ (\text{at anode}) \quad \text{(s6)}$$

$$E_3 = E^0_{\mathrm{Ag}} + \frac{RT}{2F}\ln\frac{{a_{\mathrm{Ag}^{+}}}^2}{{a_{\mathrm{Ag}}}^2} \quad \text{(s7)}$$

$$E_4 = E^0_{\mathrm{Cl}} + \frac{RT}{2F}\ln\frac{{a_{\mathrm{Cl}^{-}}}'^2}{{P_{\mathrm{Cl}_2}}'} \quad \text{(s8)}$$

The general electrolysis potentials of eqs. s1 and s2 can be derived by taking the reaction potential of eqs. s5 and s6 as a reference. The electrodeposition potential ΔE for Me based on Ag^+/Ag is given as:

$$\Delta E = E_1 - E_3 = -(E_2 - E_4) = -\frac{RT}{2F}\left(\ln\frac{{a_{\mathrm{Cl}^{-}}}^2}{P_{\mathrm{Cl}_2}} - \ln\frac{{a_{\mathrm{Cl}^{-}}}'^2}{{P_{\mathrm{Cl}_2}}'}\right) \quad \text{(s9)}$$

Assuming that the generated chlorine is pure, we have $a_{\mathrm{Cl}^{-}} = a'_{\mathrm{Cl}^{-}} = 1$, and

$$\Delta E = \frac{RT}{2F}\ln\frac{P_{\mathrm{Cl}_2}}{{P_{\mathrm{Cl}_2}}'} \quad \text{(s10)}$$

In this study, Li–Ca alloy ($a_{\mathrm{Li}} = 0.294, a_{\mathrm{Ca}} = 0.706$) precipitates in LiCl–$CaCl_2$ (65:35 mol%) at 823 K, as shown in **Fig. S1**. We consider the following reactions:

$$\mathrm{Li} + \frac{1}{2}\mathrm{Cl}_2 = \mathrm{LiCl} \quad \text{(s11)}$$

$$\mathrm{Ca} + \mathrm{Cl_2} \rightarrow \mathrm{CaCl_2} \tag{s12}$$

$$\mathrm{Ag} + \frac{1}{2}\mathrm{Cl_2} \rightarrow \mathrm{AgCl} \tag{s13}$$

Given the standard Gibbs free energy in eq. s11 as:

$$\Delta G^0(\mathrm{Li}) = -RT\ln\frac{a_{\mathrm{LiCl}}}{a_{\mathrm{Li}} \cdot P_{\mathrm{Cl_2}}{}^{0.5}} \tag{s14}$$

$$P_{\mathrm{Cl_2}}(\mathrm{Li}) = 2.696 \times 10^{-43} \tag{s15}$$

Similarly, we get from eq. s12

$$\Delta G^0(\mathrm{Ca}) = -RT\ln\frac{a_{\mathrm{CaCl_2}}}{a_{\mathrm{Ca}} \cdot P_{\mathrm{Cl_2}}} \tag{s16}$$

$$P_{\mathrm{Cl_2}}(\mathrm{Ca}) = 1.361 \times 10^{-43} \tag{s17}$$

From eq. s13, since $P'_{\mathrm{Cl_2}}(\mathrm{Ag}) = 3.832 \times 10^{-12}$, the precipitation potential of Li (29.4 mol%) was estimated by applying eq. s10 as $\Delta E(\mathrm{Li}) = -2.543$ V. Similarly, the precipitation potential of Ca (70.6 mol%) is given as $\Delta E(\mathrm{Ca}) = -2.567\ V$. Although the mixing interaction between Li and Ca was not considered in this procedure, the precipitation potentials are almost the same (about -2.55 V), while these values will be more positive in the actual system. As is clear from **Fig. S2**, Mo does not form solid solution with either Li or Ca at 823 K.

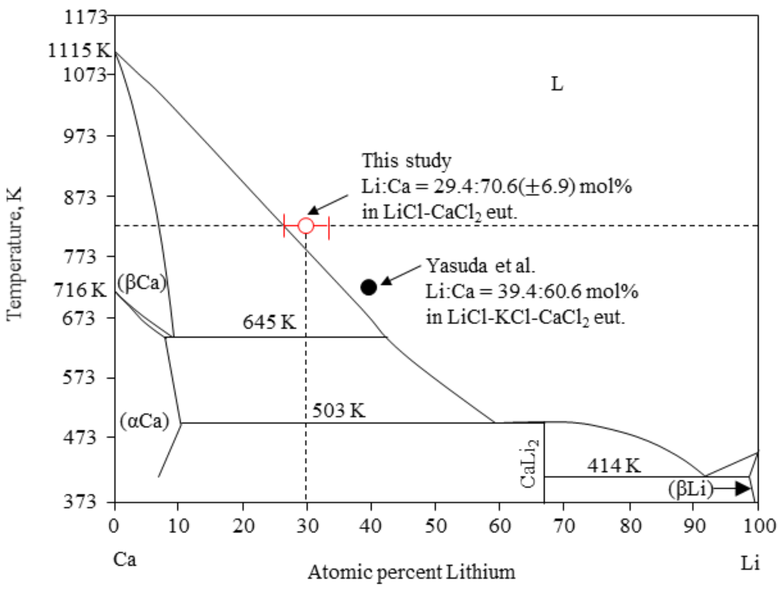

Figure S1| **Phase diagram for Li–Ca binary system. Li–Ca alloy compositions produced by molten salt electrolysis are indicated in this diagram [39].** Note that Yasuda et al. employed 52.3:11.6:36.1 mol% LiCl–KCl–$CaCl_2$ eutectic melt (m.p. 685 K) at 723 K as the electrolyte [20].

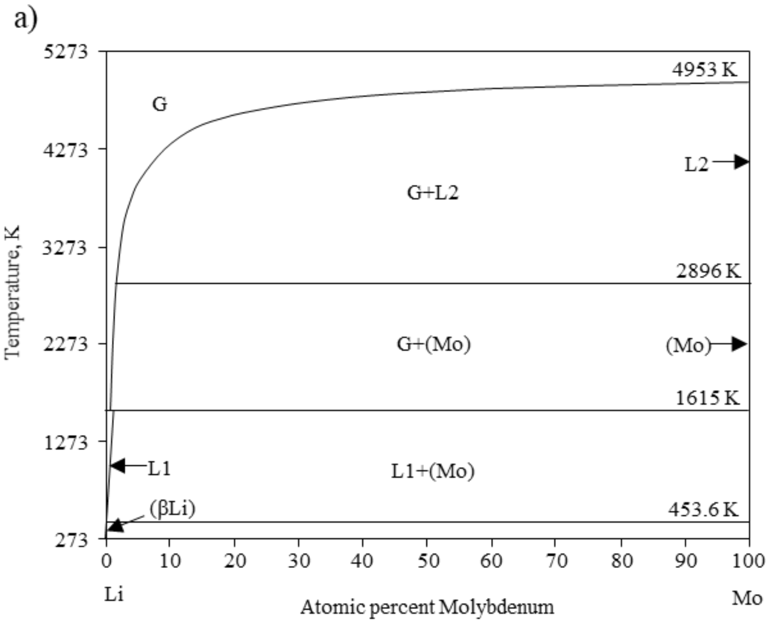

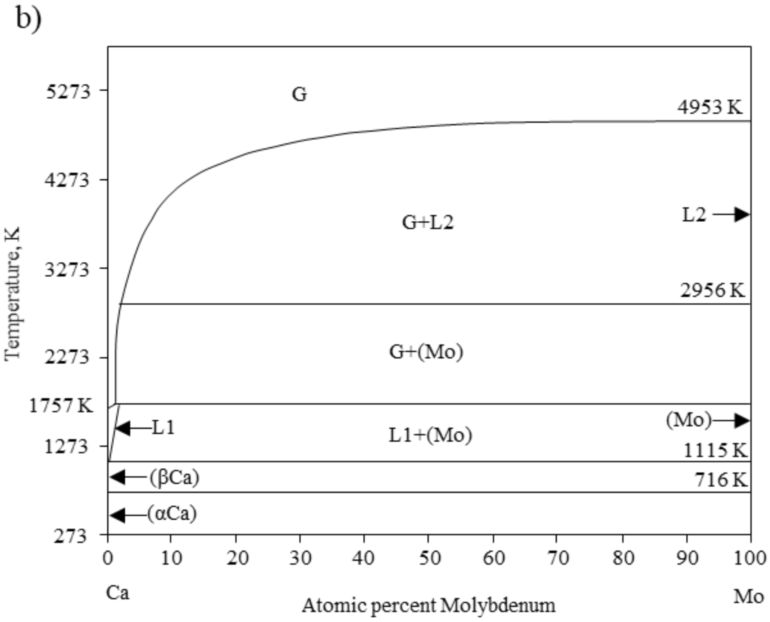

Figure S2| **Phase diagram for a): Li–Mo, and b): Ca–Mo binary systems [40].**

Appendix 2. Estimation of interfacial tension between electrodeposited Me liquid alloy and molten salt by the pendant drop method[41–44, 55,56]

The interfacial tension between liquid Me (Li_xCa_y) alloy and molten LiCl–$CaCl_2$ has not been reported as far as the authors know. For this field, it is an important work in the future to accurately measure the interfacial tension under various conditions. Here, the interfacial tension at the time of electrolytic deposition was estimated directly using a simple approach using the pendant drop method linked in our experimental system. A hanging droplet was extruded in the vertical direction from the tip of the Al_2O_3 capillary, and the interfacial tension was determined from the shape corresponding to the maximum drop volume. **Figure S3** shows a single image of the hanging droplet. Here, the d_s/d_e method gives the interfacial tension using only the curvature at the tip of the droplet [41]. Therefore, wetting of the Al_2O_3 tube by liquid Me alloy is not a problem. The interfacial tension σ can be given by

$$\sigma = \frac{\Delta\rho \mathbf{g} {d_e}^2}{\beta} \quad \text{(s18)}$$

where β is defined using the principal curvature radius, d_e, of the hanging drop at its highest point. If the density difference $\Delta\rho$ between the molten salt and the liquid Me is known, then σ can be calculated.

The physical properties of the liquid phase were determined as follows. We derived Me alloy by electrowinning, and carefully polished its surface. The alloy was dissolved in pure water, and the composition ratio between Li and Ca was determined by inductively coupled plasma–atomic emission spectrometry (ICP–AES, Thermo Scientific, ICAP–6300) using both Ca and Li standard solutions (Wako Chemical Co., Ltd., 1000 ppm). The quantitative analysis was carried out by a calibration curve method, whereby a correlation coefficient $R^2 = 0.9999$ between the spectral intensity and concentration was confirmed. Based on four measurements, the composition of the precipitated alloy was Li:Ca = 29.4:70.6(±6.9) mol%. It is difficult to accurately determine the density of this liquid alloy, but a value of $\rho_{alloy,823\,K} = 1.254 \pm 0.55\ \mathrm{kg/m^3}$ was estimated from the reported physical property data [42,43]. The density of LiCl–$CaCl_2$ eutectic salt is $\rho_{salt,823\,K} = 1.790\ \mathrm{kg/m^3}$ [44]. From eq. s18, the interfacial tension between liquid Me and molten LiCl–$CaCl_2$ is given as $\sigma_{823\,K} \approx 39\ \mathrm{mN/m}$.

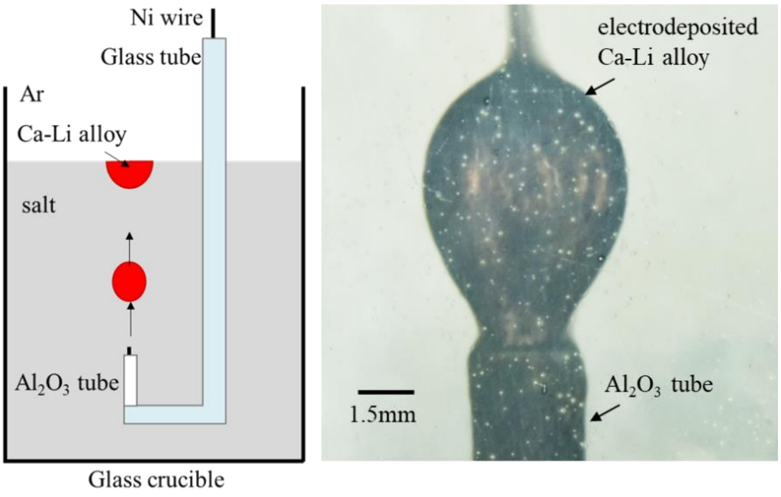

Figure S3| **Ca–Li alloy electrodeposited at −2.55 V in molten LiCl–$CaCl_2$ at 823 K.**

Appendix 3. Time change of the electrode interface

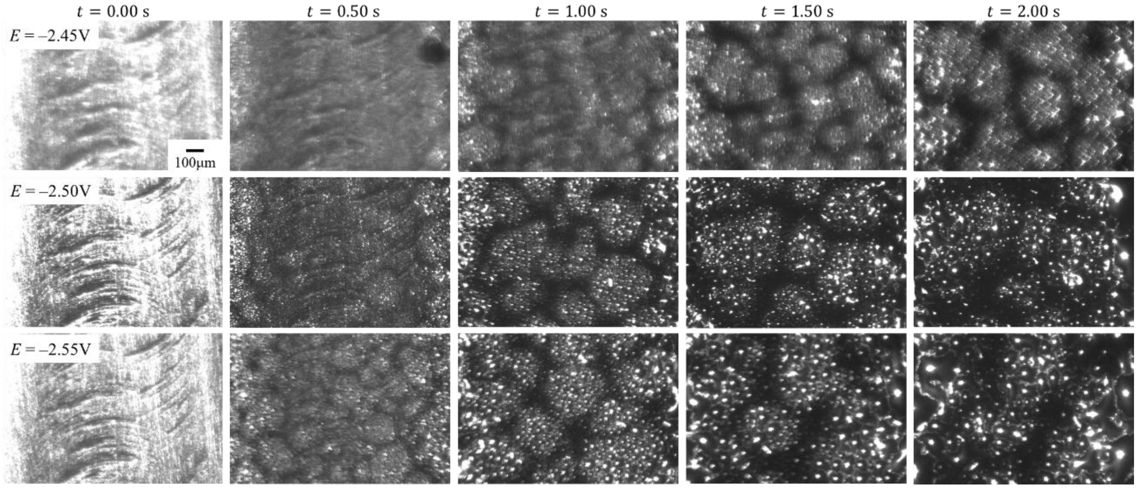

Figure S4| **Morphological change of the electrodeposited melt with time, and of black colloidal networks on the same region of flat Mo electrode at 823 K.** The snapshots were for almost the same region, and the current–time curves are represented in Fig. 1b.

Appendix 4. **Estimation of thermal behavior in the electrochemical reaction**

The thermal behavior accompanying the electrochemical reaction was described in previous studies [49–51]. Here we will briefly describe our interpretation. Firstly, when the temperature T and the pressure P are constant and the chemical reaction proceeds, according to the first law of thermodynamics, the internal energy ΔU and the heat absorbed by the reaction system Q are given as follows.

$$\Delta U = Q - P\Delta V \tag{s19}$$

$$Q = \Delta U + P\Delta V = \Delta H \tag{s20}$$

In the isobaric process, if work other than expansion/compression is denoted as W, the enthalpy change ΔH is given as follows.

$$\Delta H = T\Delta S - W \tag{s21}$$

If the chemical reaction proceeds reversibly, then

$$\Delta U = T\Delta S - (P\Delta V + W) \tag{s22}$$

In this case, W is the electrochemical work $W = {W_{elec}}^0$. Assuming that the electrochemical system is in equilibrium, the following relationship is obtained.

$$\Delta U = T\Delta S - (P\Delta V + {W_{elec}}^0) \tag{s23}$$

Thus,

$$-{W_{elec}}^0 = \Delta U + P\Delta V - T\Delta S = \Delta G \tag{s24}$$

where ΔG is the Gibbs free energy. If the electrochemical reaction is in progress, the electrochemical work should be written as $W = W_{elec}$, and we get

$$\Delta U = Q - (P\Delta V + W_{elec}) \tag{s25}$$

Therefore,

$$Q = T\Delta S + W_{elec} - {W_{elec}}^0 \tag{s26}$$

In this case, Q is the heat absorbed when the electrochemical reaction is proceeding. We consider the current I and the heat dQ absorbed during time dt. The electrochemical work is the product of the applied voltage (potential difference, ΔE) and the electric charge (Idt), and the equilibrium potential of each electrode is given as E_1^0 and E_2^0. Then we derive

$$\Delta W_{elec}{}^{0} = (E_1^0 - E_2^0)Idt \qquad \text{(s27)}$$

Here we must add the heat generation term in the right–hand side, because the Joule heat effect cannot be ignored as the electrochemical reaction proceeds. The total resistance of the system R and the potential of each electrode E_1 and E_2 are taken into account, for a unit time *dt* we get

$$\Delta W_{elec} = (E_1 - E_2)Idt - I^2Rdt \qquad \text{(s28)}$$

Therefore, the following energy equation is derived.

$$\frac{dQ}{dt} = T\Delta S + (E_1 - E_1^0)I - (E_2 - E_2^0)I - I^2R \qquad \text{(s29)}$$

According to Maeda [48,49], the heat absorbed at the single electrode/electrolyte interface dQ_e can be written as:

$$\frac{dQ_e}{dt} = \frac{T\Delta S_M I}{nF} - |E - E^0|I - I^2R_e \qquad \text{(s30)}$$

where ΔS_M indicates molar entropy change in unipolar reaction, n is the valence of ion, F is the Faraday constant, and R_e indicates resistance at electrode/electrolyte interface. In this case, the well–known Faraday's law was applied, and the transport heat of each species was ignored. The first term on the right–hand side shows the heat of chemical reaction (Q_r), the second term shows the overheat (Q_O), and the third term shows Joule heat (Q_J).

In order to conduct the experimental thermal analysis of the electrode interface, we applied the hot thermocouple method to the electrochemical system. Figure S5 shows the structure of the electrolysis cell. The K–type thermocouple (TC) was used as both the working electrode and the heat detection sensor. Another TC was placed in the solution bulk, so that the temperature difference between two TCs (ΔT) can be measured immediately after the electrochemical reaction. By adopting chronoamperometry, the potential was changed to the open–circuit potential (OCP) after applying a constant potential for a predetermined time. **Figure S6** shows the current time (*i*–*t*) curves and electrode temperature changes in 823 K molten LiCl–$CaCl_2$. The reduction current generated under the applied potential corresponds to the Li–Ca alloy formation, and the temperature difference immediately after the potential application indicates that a large amount of heat was generated during electrolysis on the electrode. As time elapses, dissolution of Li–Ca alloy and heat diffusion proceeded at OCP, then the temperature difference decreases. When the electrolysis time was relatively short, as shown in Fig. S6 a) and b), the influence on the temperature change from the magnitude of the applied potential was not very large, because there was not enough time to cause the temperature change. In Fig. S6 c), the temperature change varied greatly according to the applied potential. Thus, the relationship between the amount of electrochemical reaction and the heat transfer becomes clear by measuring the temperature change after a constant current flows for a predetermined time. In Fig. S6

d), at $E = -2.60$ and -2.55 V, the maximum temperature difference of $\Delta T \approx 45\ K$ appeared for a fixed time immediately after the electrolysis and then it decreased. From the above measurement, there was a temperature difference over 10 K even when the potential was applied for as short as 1.00 s. Separation of the contribution from Q_r, Q_O, and Q_J to this temperature difference is not feasible at the present time and will be discussed in our next report. The temperature gradient of surface tension between "pure Ca" and "pure Li" is reported as 0.10 to 0.15 mN m^{-1} K^{-1}. [52]

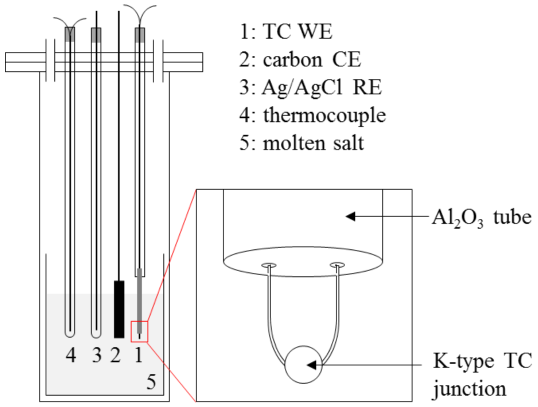

Figure S5| **Schematic diagram of the cell for thermal measurement of the electrochemical reaction.** Focused electrochemical reaction proceeds at the surface of TC working electrode.

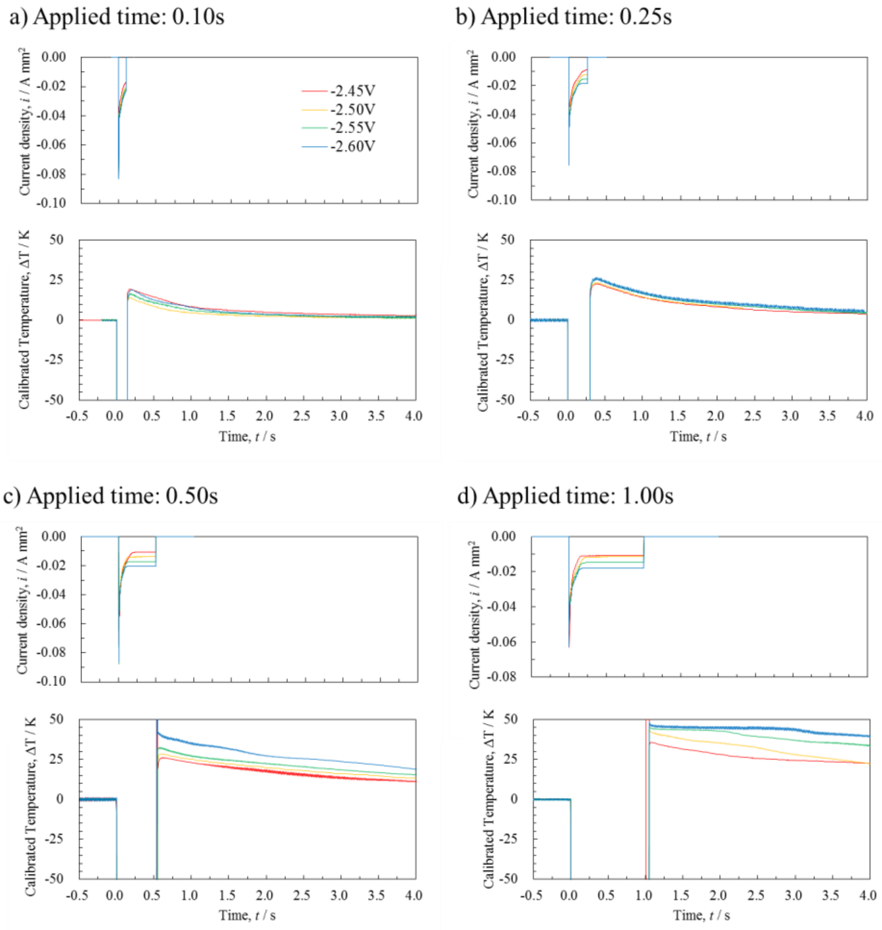

Figure S6| ***i*–*t* and Δ*T*–*t* relations of K–type thermocouple in LiCl–$CaCl_2$ eutectic melt at 823 K.**

Appendix 5. Experimental apparatus

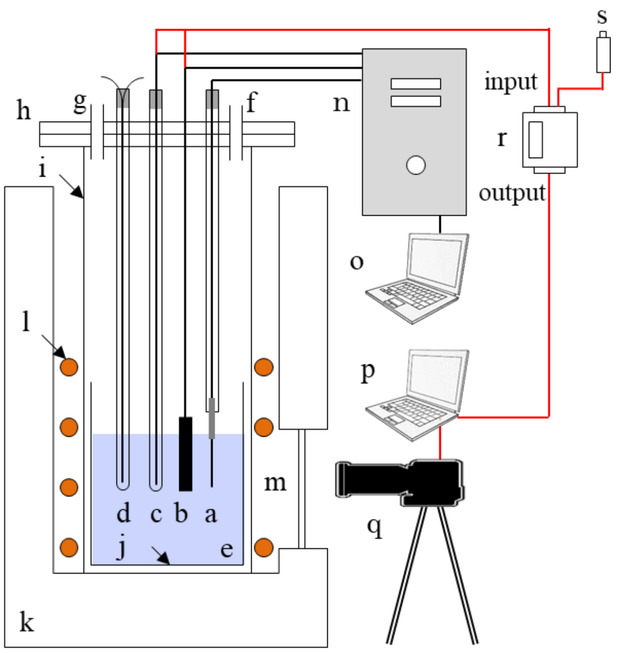

a: Mo rod WE
b: carbon rod CE
c: Ag/Ag^{+} RE
d: thermocouple
e: LiCl-$CaCl_2$ eutectic melt
f: Ar inlet
g: gas outlet
h: sealed flange
i: quartz vessel
j: glass crucible
k: refractory
l: SiC heater
m: quartz window
n: polarization system
o: computer 1
p: computer 2
q: digital microscope
r: synchronization system
s: trigger

Figure S7| **Schematic diagrams of the experimental setup.** (black line: wiring related to electrolysis, red line: wiring related to high–speed imaging).
